# *Lactococcus lactis* expressing sand fly PpSP15 salivary protein confers long-term protection against *Leishmania major* in BALB/c mice

**DOI:** 10.1371/journal.pntd.0007939

**Published:** 2020-01-03

**Authors:** Elaheh Davarpanah, Negar Seyed, Fariborz Bahrami, Sima Rafati, Reza Safaralizadeh, Tahereh Taheri

**Affiliations:** 1 Department of Biology, Faculty of Natural Sciences, University of Tabriz, Tabriz, Iran; 2 Department of Immunotherapy and *Leishmania* Vaccine Research, Pasteur Institute of Iran, Tehran, Iran; 3 Department of Immunology, Pasteur Institute of Iran, Tehran, Iran; Universidade Federal de Minas Gerais, BRAZIL

## Abstract

Cutaneous leishmaniasisis a vector-borne disease transmitted by *Leishmania* infected sand flies. PpSP15 is an immunogenic salivary protein from the sand fly *Phlebotomus papatasi*. Immunization with PpSP15 was shown to protect against *Leishmania major* infection. *Lactococcus lactis* is a safe non-pathogenic delivery system that can be used to express antigens *in situ*. Here, the codon-optimized *Ppsp15-egfp* gene was cloned in pNZ8121 vector downstream of the PrtP signal peptide that is responsible for expression and secretion of the protein on the cell wall. Expression of PpSP15-EGFP recombinant protein was monitored by immunofluorescence, flow cytometry and Western blot. Also, expression of protein in cell wall compartment was verified using whole cell ELISA, Western blot and TEM microscopy. BALB/c mice were immunized three times with recombinant *L*. *lactis*-PpSP15-EGFP^cwa^, and the immune responses were followed up, at short-term (ST, 2 weeks) and long-term (LT, 6 months) periods. BALB/c mice were challenged with *L*. *major* plus *P*. *papatasi* Salivary Gland Homogenate. Evaluation of footpad thickness and parasite burden showed a delay in the development of the disease and significantly decreased parasite numbers in PpSP15 vaccinated animals as compared to control group. In addition, immunized mice showed Th1 type immune responses. Importantly, immunization with *L*. *lactis*-PpSP15-EGFP^cwa^ stimulated the long-term memory in mice which lasted for at least 6 months.

## Author summary

Different strains of *Lactococcus lactis* can be used as a suitable non-pathogenic vehicle for live vaccination. In this study, our results demonstrated that recombinant *L*. *lactis* could express PpSP15, an immunogenic component of *Phlebotomus papatasi* saliva, on the cell wall. Furthermore, localizing PpSP15 on the cell wall of *L*. *lactis* could trigger short (2 weeks) and long (6 months) memory cellular immunity in BALB/c mice, immunized with the recombinant *L*. *lactis*. In addition, after challenging the immunized mice with *Leishmania major*, the cytokine assays indicated that their immune response was shifted to Th1 which controlled the footpad swelling and propagation of *L*. *major* in the lymph nodes.

## Introduction

Leishmaniasis are high-prevalence parasitic diseases with a long history in the world [1]. Theses group of diseases are exhibited in different forms including cutaneous (CL), mucocutaneous (MCL) and visceral (VL) leishmaniasis [2]. All forms of leishmaniasis lack an effective treatment (mostly due to drug resistance) and a protective vaccine, in spite of many efforts by the researchers in recent decades [3].

The main route of parasite transmission to humans is through biting by female sand flies [4]. Metacyclic promastigotes are regurgitated by sand flies during the blood feeding process. During this process the sand fly also delivers saliva at the infection site. Sand fly saliva contains bioactive proteins including anticoagulants, inhibitors of platelet aggregation and anti-complement molecules among other biological activities [5]. Some of these bioactive have immunomodulatory effects in the host [6, 7]. Importantly, some of these salivary proteins are immunogenic and can elicit a host immune responses [8]. The sand fly salivary protein PpSp15 was characterized as an immunogenic protein [9–12]. Furthermore, immunization with *P*. *papatasi* PpSP15 was shown to be protective against *L*. *major* infection [13] by inducing a cellular immune response in a form of a delayed type hypersensitivity (DTH) response [14].

To design a successful vaccine an immunogenic antigen must be chosen from a pathogen together with a suitable delivery system. The selected antigen should trigger also a long-term immunity [15, 16]. Common weaknesses of most delivery systems are their instability, degradation inside the cells and low expression of their delivered antigens [17]. Suitable live delivery systems include *Brucella abortus*, *Salmonella enterica*, *Listeria monocytogenes*, *Bacillus subtilis*, *Lactobacillus plantarum*, *Lactococcus lactis (L*. *lactis)* and *Leishmania tarentolae*. These systems offer more stability, amplification, production of heterologous antigens into the cell in natural form and, most importantly, *in vivo* stimulation of the immune system [18, 19]. The advantages of *L*. *lactis* (NZ9000 strain) compared with other non-pathogenic expression systems are its less endogenous and no exogenous proteases, being LPS-free, and lack of inclusion bodies and spores [20, 21]. In addition, *L*. *lactis* for long has been safely used in dairy products [22–24]. Proteoglycan compounds in the cell wall of this bacterium may exhibit adjuvant effects; hence, they can contribute to the immune response stimulation [23, 25]. This bacterium has been used as one of the best delivery tools to express and transmit bioactive molecules [26]. *Lactococcus lactis* has also been widely used as a suitable delivery system for vaccines or for *in vivo* production of heterologous therapeutic proteins [27–30]. The application of *L*. *lactis* in vaccine designs against various diseases confirms its effectiveness as a suitable carrier for antigens [31–33].

Furthermore, the rapid assessment of the expression of heterologous proteins is technically essential for the downstream studies [34]. In the absence of a specific antibody against a desired protein, EGFP reporter helps to identify the expressed protein through different tools such as ELISA, Western blot, direct fluorescent microscopic observation and flow cytometry [35].

In the present study, we used *L*. *lactis* as a live expression system to express the PpSP15 protein infused with EGFP on the surface of the bacteria. BALB/c mice vaccinated with this system were challenged with *Leishmania major* and the type of immune response was measured as well as the short (2 weeks) and long term (6 months) protection of this vaccine.

## Materials and methods

### BALB/c mice and ethics statement

In all experiments, female BALB/c mice (6–8 weeks old, weight range 17±1 g) were purchased from Pasteur Institute of Iran animal breeding facilities and were kept in plastic cages under standard conditions (12 h light-dark cycle, 23–28°C temperature, 50–60% humidity with normal rodent diet). This study was designed according to the regulations of the Animal Research Ethics Committee of Pasteur Institute of Iran, which are revised by the latest version of the Specific National Ethical Guidelines for Biomedical Research (MOHME-2005).

### Gene cloning, induction and expression of protein

Two genes, *Ppsp15-egfp* (~1127 bp, with a 15 nt as a linker) and *egfp* (~746 bp), were codon optimized to be expressed in *L*. *lactis* and synthesized (Biomatik) in pBluescript II SK (+). Both constructs were digested by *Eco*RV/*Xba*I restriction digestion enzymes (Mannheim), purified from the agarose gel by kit (Promega) and cloned in the same site in pNZ8121 vector (Mobitec) containing a nisin-inducible promoter (PnisA) and chloramphenicol resistance gene (Cm^R^). After heat-shock transformation into *E*. *coli* strain MC1061 (Mobitec), the bacteria were plated on LB (Luria-Bertani) agar+5 μg/ml Chloramphenicol (Applichem) and incubated at 37°C. After sequence confirmation of both genes into the pNZ8121 vector, ~500 ng of each (pNZ8121-*Ppsp*15-*egfp*^cwa^ and pNZ8121-*egfp*^cwa^) were transferred into competent *L*. *lactis* strain NZ9000 (Mobitec) through electroporation (Biorad Gene pulser) in a pre-chilled cuvette 2 mm (adjustment: 2000 V, 25 μF, 200 Ω). Transformed *L*. *lactis* were incubated in M17 Broth (Difco) supplemented with 2.5% Glycine (Sigma), 0.5 M Sucrose (Merck), 20 mM MgCl_2_ and 2 mM CaCl_2_ for 1–1.5 h at 30°C and then plated on M17 agar+0.5% Glucose (Sigma)+5 μg/ml Chloramphenicol. After 48 h incubation at 30°C without aeration, clones appeared.

To induce expression, overnight cultures of both recombinant *L*. *lactis* were diluted (∼1/100) in fresh media and grown until OD_600_ = 0.4–0.5 and induced with NICE Nisin kit (1–2 ng/ml Nisin, 5% acetic acid, Mobitec). Expression of both proteins in different times after induction was confirmed through several methods containing Western blot, ELISA, flow cytometry and microscopy monitoring.

### Cell fractionation

Total protein extracts from different cell compartments (cell wall, cytoplasmic, and membrane) were obtained by an adjusted method of Y. Dieye et al. [36]. The pellet of induced bacteria was lysed in TES buffer (10 mM Tris-HCl pH 8, 1 mM EDTA, 25% Sucrose, all from Sigma) plus lysozyme (5 mg/ml, Boehringer, Mannheim). After 1 h incubation at 37°C, protoplasts were pelleted by centrifugation (4300 x*g*/10 min/4°C). Then, supernatant containing cell wall proteins was concentrated by 100% trichloroacetic acid (TCA) and incubated for 30 min on ice, then precipitated with 16000 x*g*/ 10 min at 4°C [37]. The pellets of protoplasts were washed with TES buffer and resuspended in 500 μl of sterile dH2O. The suspension was freeze-thawed for five times. Next, membranes were recovered by centrifugation at 16000 x*g*/1 h at 4°C. Supernatant that contained the cytoplasmic proteins was concentrated by TCA (Trichloroacetic acid).

### Western blot

Suspension of the log-phase of two induced recombinant *L*. *lactis* were centrifuged at 1100 x*g* for 10 min and the pellet was used for Western blot as described by Katebi, et al. [10]. Briefly, the pellet was lysed in sample buffer and resolved by 12.5% SDS-PAGE. After transferring the protein bands from the gel onto a nitrocellulose membrane (Schleicher and Schuell Bioscience), the membrane was incubated overnight into blocking solution (2.5% BSA/0.1% Tween20 in TBS solution (10 mM Tris–HCl (Sigma), pH 7.4, 150 mM NaCl). Then, membranes were probed with diluted anti-GFP (Green fluorescent protein) Ab (polyclonal antibody to GFP-HRP; Acris antibodies GmbH) 1:5000 in blocking solution. Finally, the DAB powder (Sigma) solubilized in 50 mM Tris–HCl, pH 7.4 along with 0.01% (v/v) H_2_O_2_ (Sigma) were used as a substrate [38].

### Whole cell ELISA

For whole cell ELISA, nisin induced live- or heat-killed- (1 h in boiling water) bacteria in PBS (10^9^ CFU/ml) or supernatant of each were coated in 96-well maxi sorb plate (SPL) in duplicate and incubated overnight at 4°C. Before and after each step, washing was done three times with PBS plus 0.05% Tween20. To prevent nonspecific bindings, blocking was performed with 1% BSA in PBS for 2 h at 37°C. Then, polyclonal anti-GFP Ab conjugated with HRP diluted (1:10000) in PBS/ 1% BSA/ Tween20 and incubated for 2 h at 37°C. The Peroxidase Substrate System (KPL, ABTS) was added and incubated for 30 min. Then, stop reagent (SDS 1%) was added to each well and absorbance read at 405 nm by ELISA reader (Sunrise, Tecan) [39].

### Flow cytometry and fluorescence microscopy

In the same way, 3 hours and overnight induced bacteria were assessed for EGFP expression in *L*. *lactis*-PpSP15-EGFP^cwa^, *L*. *lactis*-EGFP^cwa^ (as a positive control) and *L*. *lactis* (as a negative control) using a FACS caliber flow cytometer equipped with a 488-nm laser. For each sample, 50,000 cells were counted.

For Fluorescence microscopy, three lines of bacteria were centrifuged at 3000 rpm for 10 min and resuspended in PBS. The bacterial pellet was observed directly using fluorescence microscopy (emission at 490–530 nm filter, Nikon, E 200, ACT-1 software, Digital sight Camera) with a 100x magnification.

### Transmission electron microscopy (TEM)

For transmission electronic microscopy (TEM), 3 h after induction, *L*. *lactis*-PpSP15-EGFP^cwa^ with Nisin and non-induced *L*. *lactis* were washed in PBS and suspended in the first fixator solution (3% glutaraldehyde in 0.1 M PBS at pH 7.2) and incubated in room temperature for at least 30 min. Then, samples were washed 3 times using PBS and then suspended in the second fixator (1% osmium tetroxide in PBS at pH 7.2) for 2 h at room temperature. After three times washing, dehydration was done with acetone and ethanol. The samples were then fixed by three times incubations for 16 h at 4°C in 50, 75 and 100% Spurr resin in ethanol, respectively. After polymerization of the resin (70°C, 48 h), ultra-thin sections (60 nm) were cut and examined with TEM (Ziess EM900) [40].

### Toxicity of bacteria in BALB/c mice

Both recombinant bacteria (*L*. *lactis*-EGFP^cwa^ and *L*. *lactis*-PpSP15-EGFP^cwa^) and wild type *L*. *lactis* were washed with apyrogenic PBS and suspended in 100 μl PBS. Each bacterial preparation was injected into the tail vein of the 3 BALB/c mice, separately (intravenously, ~2×10^9^ CFU). Body temperature, weight and physical movements of the mice were monitored up to 120 h after bacteria injection [41].

### Mice immunization and challenging

Different groups of BALB/c mice (S1 Table, 12 mice/group) were immunized subcutaneously (s.c.) three times with *L*. *lactis*-PpSP15-EGFP^cwa^ (G1) as a main group or *L*. *lactis*-EGFP^cwa^ (G2) as a control group in the right hind footpad in the same regimen in 2-weeks intervals (at 0, 2 and 4 weeks). All immunizations were done with ~2×10^9^ CFU of bacteria in 50 μl PBS. G3 (PBS) was remained without immunization as a control. After the last immunization, all groups were divided into two categories. First category was challenged at 2 weeks after last immunization to test short-term (ST) protection, and the second category remained without challenging for at least six months to evaluate long-term (LT) protection assay. All groups were infected s.c. in the left hind footpad with metacyclic form of promastigotes *L*. *major* (MRHO/IR/75/ER) ~2×10^5^/mouse plus salivary gland homogenate (SGH derived from *P*. *papatasi*, provided by Dr. Shaden Kamhawi and Dr. Jesus G. Valenzuela) 0.5 pair/mouse. The promastigote of *L*. *major* was cultured in M199 medium (Sigma) supplemented with 5% heat-inactivated fetal calf serum (hi-FCS, Gibco), 40mM HEPES (Sigma), 1mM L-glutamine (Sigma), 0.1mM Adenosine (Sigma), 0.5μg/ml Hemin (Sigma) and 100μg/ml Gentamicin (Biosera). The stationary-phase metacyclic form was isolated from stationary phase of parasite after 5 passages *in vitro* through gradient Ficoll 400 (Sigma) and washed in PBS. Footpad thickness in infected mice was measured weekly by a digital caliper (resolution: 0.01 mm).

### Limiting dilution assay

To estimate the parasite number after infection, 4–6 mice per group were randomly selected and the popliteal lymph nodes (LNs) extracted, weighed, and homogenized in Schneider’s Drosophila medium (Sigma) enriched with 10% hi-FCS plus 50 μg/ml Gentamicin (Biosera). The cell suspension serially was diluted 1:5 (from 10^−1^ to 10^−15^) and transferred into two wells of flat bottom 96-well plates (Orange scientific). Growing of parasite in all wells was checked microscopically during 14 days. The well number containing even one live promastigote was recorded and the parasite burden computed using the following formula: -Log_10_ (last dilution with live parasites/weight of homogenized LN) [42].

### Cellular immune response and cytokine assay

The derived spleens from 4–6 mice per group were separately homogenized and exposed 5 min with ACK lyses buffer (0.15 M NH_4_Cl, 1 mM KHCO_3_ and 0.1 mM Na_2_EDTA) for erythrocyte cell lysis. Splenocytes were washed with un-supplemented culture media and final pellet resuspended in phenol- red free DMEM (Sigma) supplemented with 10% hi-FCS, and counted. The cells (~3.5×10^6^ cells/ml) were plated with different antigens, including: *L*. *major* F/T (20 μg/ml), Concanavalin A (5 μg/ml) as a positive control and without any antigen as a negative control and kept at 37°C in 5% CO_2_ humidified incubator. The supernatant was collected 3 days after stimulation for measuring IL-5 and IL-17 and 5 days for determination level of IFN-γ and IL-10. Cytokines productions were measured using sandwich ELISA DuoSet R&D kits (R&D) according to the manufacturer’s protocols.

### Statistical analysis

Statistical analysis was accomplished using Graph-Pad Prism 6.0 for Windows (La Jolla). All data are presented as mean±SD and the statistical comparison of the two data-sets was calculated using Mann-Whitney U test. P values less than 0.05 were considered significant. The whole *in vivo* experiment was done and completed two times independently. All *in vitro* experiments were repeated at least two times.

## Results

### Generation of two constructs and protein expression

Two desired genes were cloned downstream of the PnisA promoter and the PrtP signal peptide for protein attachment on the cell wall in pNZ8121 to produce two constructs (pNZ8121-*Ppsp*15-*egfp* and pNZ8121-*egfp*, S1A and S1B Fig.) Expression of PpSP15-EGFP (~42 kDa) and EGFP (~27 kDa) proteins after induction were confirmed through different methods, namely Western blotting (S1C Fig), fluorescent microscopy (S1D Fig) and flow cytometry (S1E Fig). Different nisin concentration (2, 5 and 10 ng/ml) and also different incubation times after the induction (3 h and overnight) were verified to find the best conditions. The concentration of 2 ng/ml nisin and 3 h after the induction were chosen to prepare CFU.

Expressions of both recombinant preparations of *L*. *lactis* were verified using Western blot with the recombinant EGFP (rEGFP) was used as a positive control. The specific bands for both expected proteins, namely EGFP and PpSP15-EGFP, clearly confirmed their expression (S1C Fig). Furthermore, fluorescent microscopy showed both recombinant bacteria (unlike of the wild type line) could express the EGFP at least 3 h after the induction (S1D Fig). This verification by flow cytometry at 3 h after the induction confirmed that the least intensity for *L*. *lactis*^WT^ was 0.88%, and a range between 31.86% and 42.37% was obtained for *L*. *lactis*-PpSP15-EGFP^cwa^ and for *L*. *lactis*-EGFP^cwa^ as a positive control, the range of intensity was 64.67–72.11% (S1E Fig shows results from one experiment). Also, in both recombinant bacteria, the stability of two expressed proteins was confirmed for several times after freezing and re-culturing through Western blotting, observation with fluorescent microscope and ELISA.

### Confirmation of protein expression on the cell wall of *L*. *lactis*

**Fig 1:**
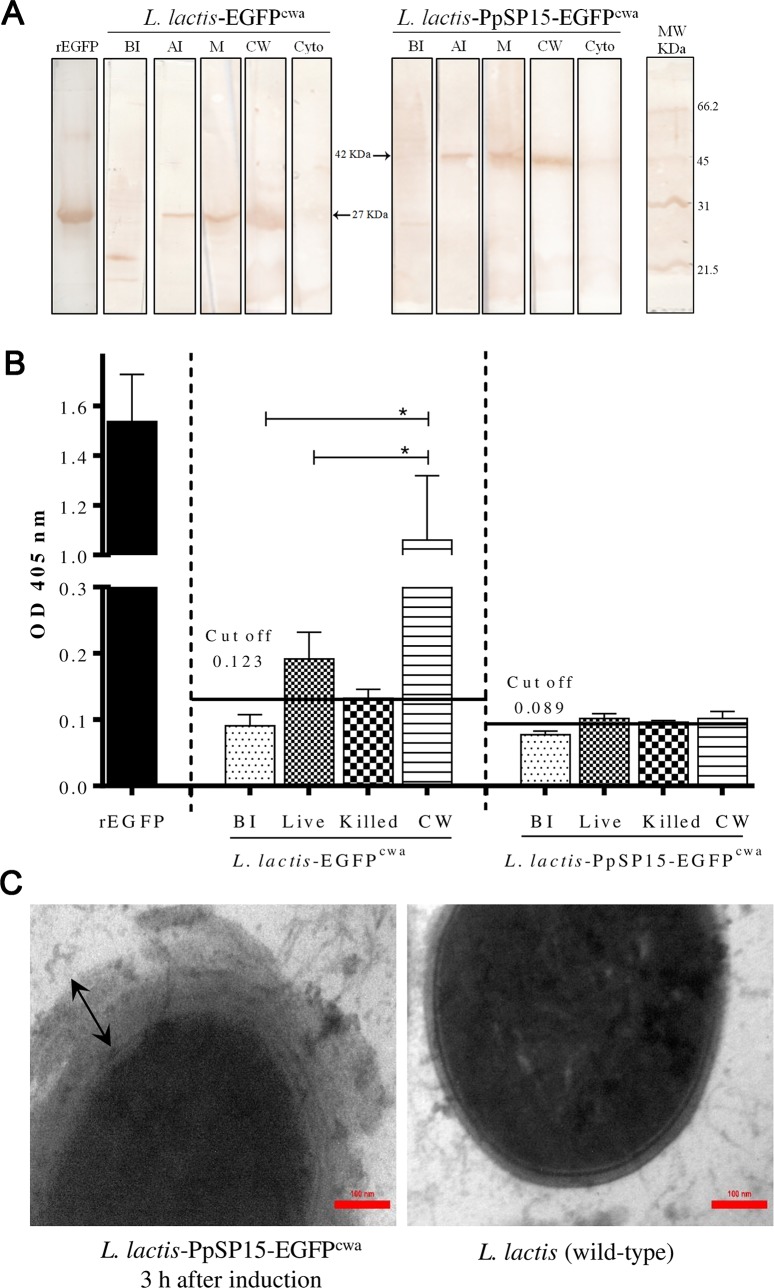
Confirmation of cell wall protein expression using several methods. (A) By Western blot analysis using different cellular compartments by anti-GFP Ab, expression of PpSP15-EGFP and EGFP proteins was shown in membrane and cell wall fraction of two recombinant bacteria, *L*. *lactis*-PpSP15-EGFP^cwa^ and *L*. *lactis*-EGFP^cwa^. BI: Before induction, AI: After induction, M: Membrane, CW: Cell wall fraction and Cyto: Cytoplasmic fraction of bacteria. The rEGFP used as a positive control that showed a super band ~27 KDa. (B) By ELISA, measurement of expression of two cell wall PpSP15-EGFP and EGFP proteins in cell wall fractionation, and different status of bacteria using anti-GFP Ab. BI: before induction (as a negative control); intact live bacteria; heat-killed cell; and CW: cell wall. Cut off = mean (BI) + 2SD. The rEGFP used as a positive control and showed the highest absorbance. *Present statistical differences between different status of bacteria. (C) Using TEM, microscopic micrographs (magnification ×50,000) of the *L*. *lactis*-PpSP15-EGFP^cwa^ (left) 3 h after induction and *L*. *lactis* wild-type as a negative control (right) clearly showed the cell wall expression of the PpSP15-EGFP on the surface of bacteria. The scale bar represents 100 nm. Two-headed arrows show the thickness on the cell wall of *L*. *lactis*-PpSP15-EGFP^cwa^.

Cloning of *Ppsp15-egfp* gene in pNZ8121 after PrtP signal peptide caused the protein expression on the cell wall of bacteria. Therefore, the expression of the protein at its final destination on the cell wall should be confirmed. As shown in Fig 1A, the results of Western blotting showed that the two PpSP15-EGFP and EGFP proteins in two different cell fractions including the membrane and the cell wall compartments were detectable. The faint bands were visible in the cytoplasm fractions in both bacteria, perhaps caused by the expression of the protein, firstly inside the cytoplasm. The rEGFP was used as a positive control.

For further clarification, the cell wall expression was confirmed by whole cell ELISA. Coating the plates with the cell wall (CW) fraction of *L*. *lactis*-EGFP^cwa^ after the induction was shown that this bacterium could express the EGFP significantly (1.06±0.26 nm, *p*<0.05) compared to the other forms (before induction (BI), intact live and heat-killed). Also, live intact *L*. *lactis*-EGFP^cwa^ indicated EGFP expression was non-significant (0.19±0.04 nm) and higher than the cut-off (mean (BI)±2SD = 0.123) compared to the heat-killed forms. Nevertheless, the cell wall fraction of *L*. *lactis*-PpSP15-EGFP^cwa^ or the intact live cells formed higher than the cut-off signal (mean±2SD = 0.089) which was not significant (Fig 1B). We used anti-GFP antibody because EGFP fused with PpSP15 could decrease the possibility of EGFP identification. We also had this concern for fluorescent microscopy (S1D Fig) and flow cytometry (S1E Fig). To verify that both Western blotting and ELISA systems were working, the purified rEGFP protein was used as a positive control and as expected, it was detected by anti-GFP-antibody in the super band and the highest absorbance (1.537±0.19 nm), respectively.

Unlike results obtained from Western blotting and ELISA for the localization of EGFP protein expressed on the cell wall, ELISA did not confirm the expression and presence of PpSP15-EGFP on the cell wall of the bacteria. To verify the expression of PpSP15-EGFP protein on the cell wall of the bacteria with more precision, *L*. *lactis*-PpSP15-EGFP^cwa^ was directly observed by TEM (Fig 1C). For this, the cell wall thickness of the two bacteria, *L*. *lactis*-PpSP15-EGFP^cwa^ (3 h after induction) and wild type *L*. *lactis* (as a negative control), were compared. The observation through TEM showed that most cells were not degraded. According to TEM micrographs (Fig 1C), there was a clear difference in thickness of the cell wall between the two bacteria. Interestingly, TEM observation clearly showed the expression of PpSP15-EGFP protein on the cell surface. The cell wall thickness in induced *L*. *lactis*-PpSP15-EGFP^cwa^ was more than 100 nm, whereas in wild type *L*. *lactis* it was less than 100 nm. Hence, the successful expression of both proteins (i.e. PpSP15-EGFP and EGFP) on the cell wall of the bacteria after the induction was confirmed by two methods.

### Toxicity evaluation of recombinant *L*. *lactis* in BALB/c mice

To study any harmful effects or toxicity of the genetically modified bacteria, different groups of BALB/c mice were infected with different lines of recombinant or wild type *L*. *lactis*. At few hours after the injection (1, 3, 5, 7, 9, 24, 72 and 120 h), all groups showed a slightly decreasing weight, and after 24 h regained the normal weight. After 24 h, there was no significant difference between groups in weight, body temperature, or behavior of mice (S2 Fig). The mice were monitored for two weeks, and there were no significant differences between them.

### Short- and long-term follow up of immunized BALB/c mice after challenge with *L*. *major*

**Fig 2:**
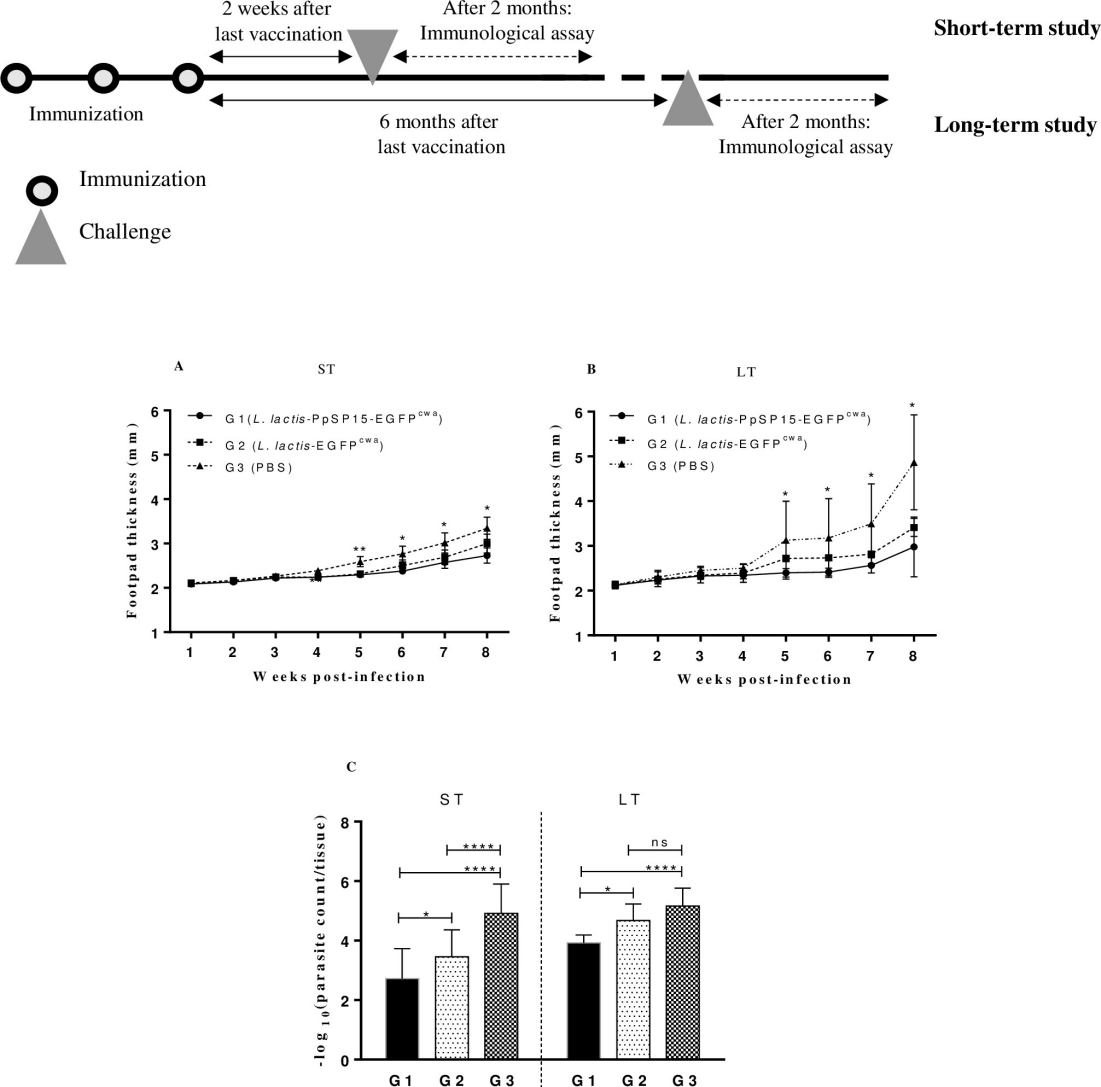
Footpad thickness and parasite burden measurment in BALB/c mice at 2 months after challenge. Schematic figure shows the time schedule for immunization, challenge and analysis during the short- and long term study. Two weeks (ST) or 6 months (LT) after immunization, all groups of BALB/c mice were infected with *L*. *major*+SGH, and development of infectivity and effectivity of *L*. *lactis*-based vaccine was evaluated through footpad thickness and parasite burden measuring. Footpad thickness in both experiments, ST (A) and LT (B), was meseaured weekly during 8 weeks after challenge by digital caliper. Two months after challenge, LNs from 4–6 mice were isolated, homogenized and after serially culturing the dilutions into the 96-wells plates monitored through weekly microscopical observation to find last dilution containing at least one parasite (C). *Present the statistical differences between different mice groups. * = p<0.05, **** = p<0.0001, ns = non-significant.

Following the assessment of cytokine profile after three immunizations and showing the immune response was toward Th1 response, all three groups of BALB/c mice were challenged with *L*. *major* plus SGH. Two major hallmarks of the infectivity rate evaluation in mice models are measuring the footpad size and estimating the parasite burden. Weekly measuring footpad swelling (as a routine method) showed a significant difference (*p*<0.05) between the immunized (G1, *L*. *lactis*-PpSP15-EGFP^cwa^) and the control groups (G3, PBS) from week 4 post-infection in ST experiment. Although the group immunized with *L*. *lactis*-EGFP^cwa^ (G2) also had less swelling in the footpad than the control G3 (PBS), there was no significant difference between them (there was a significant difference in 4^th^ and 5^th^ weeks between G2 and G3) (Fig 2A). In the long-term experiment, 8 weeks post-infection there was a slight increase in footpad thickness in G1 compared with the control groups. In addition, the footpad measurement showed significant differences (*p*<0.05) between G1 and G3 from 5^th^ to 8^th^ week and also, between G1 and G2 at weeks 6 and 7 (Fig 2B).

To assay immunization effects of *L*. *lactis*-PpSP15-EGFP^cwa^ on mice, measuring parasite amount in popliteal LNs after immunization is critical. Therefore, 2 months after challenge in both short and long-term experiments, parasite burden in popliteal LNs of the mice was estimated by limiting dilution assay and monitoring microscopic promastigotes (Fig 2C). These results in the ST experiment showed that G3 as a non-immunized control group has significantly (*p*<0.05) the most parasite in the LNs (4.92±0.98 P) than G1 (2.72±1.0 P) and G2 (3.45±0.9 P). Assessement of parasite burden in LT experience also revealed a comparable difference between G1 (3.92±0.26 P) and the other groups (4.67±0.55 P in G2 and 5.16±0.6 P in G3). Importantly, in both experiments, G2 had significantly more parasite numbers in LNs than G1.

### Short- and long-term cytokine profile after challenge with *L*. *major*

**Fig 3:**
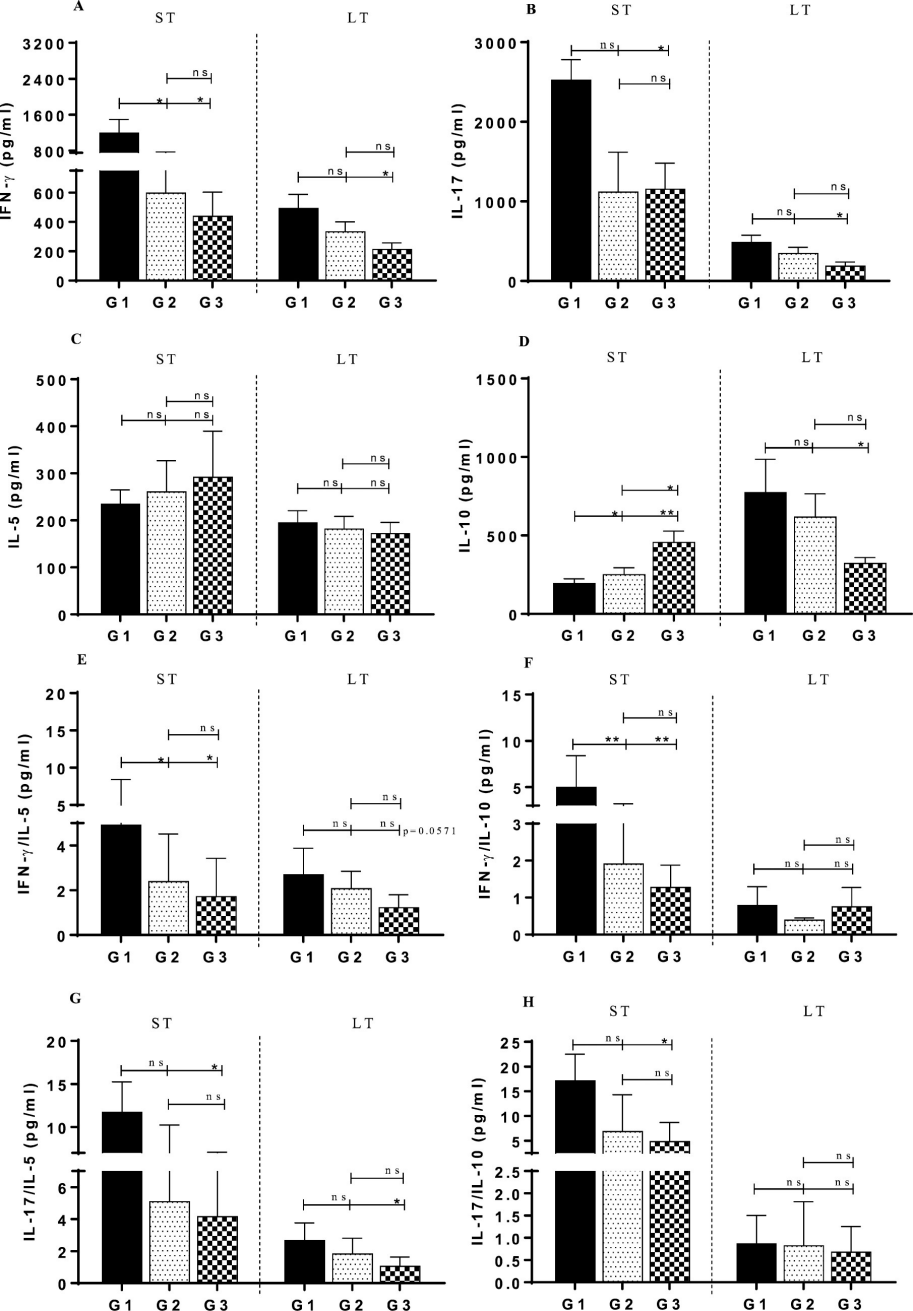
Determination of cytokine profile in both short-term (ST) and long-term (LT) experiments after infection with *L*. *major*+SGH. Two months after challenge with *L*. *major*+SGH, the cellular immune response in BALB/c mice through determination of the level of generated cytokines was compared between different groups in both ST and LT experiments. Individual splenocytes were re-estimulated with *L*. *major* F/T as antigen, and supernatants collected and analyzed for production of different cytokines. The results are depicted individually for each cytokine (A (IFN-γ), B (IL-17), C (IL-5) and D (IL-10)) or ratio between two different cytokines (E (IFN-γ /IL-5), F (IFN-γ /IL-10), G (IL-17/IL-5) and H (IL-17/IL-10)). *Present the statistical differences between different mice groups. * = p<0.05, ** = p<0.001, ns = non-significant.

In both experiments (ST and LT memory), 2 months after challenging the BALB/c mice with *L*. *major*+SGH, the productions of the cytokines were evaluated and compared between different groups. As shown in Fig 3A, after stimulation of G1 splenocytes with *L*. *major* F/T antigen, levels of IFN-γ in this group (1190.03±910.83 pg/ml in ST and 493.21±190.14 pg/ml in LT) were significantly higher than G3 in both experiments. Although IFN-γ level declined in LT, there was still a similar pattern like in ST, and G1 had the highest level among the two control groups. More interestingly, IL-17 measurement after induction with parasite antigen in ST experiment showed that G1 significantly produced higher IL-17 than the two control groups (*p*<0.05) (Fig 3B). Similar to IFN-γ, although IL-17 quantity reduced two months after the infection in LT experiment, this cytokine was still significantly higher than in G3. Furthermore, after re-stimulating splenocytes with this antigen, the level of IL-5 in all three groups was the same in both experiments (Fig 3C). Nevertheless, production of IL-5 in G1 in LT was 1.2-fold less than in G1 in ST, which can affect the shifting of the immune system to Th1 pathway. Also, after induction with *L*. *major* F/T, G1 showed a decrease in IL-10 level when compared to the other two control groups in ST experiment (Fig 3D). There were significant differences between G1 and groups 2 and 3 (*p*<0.05). Whereas the level of this cytokine in the LT in G1 was significantly higher than in G3 (*p*>0.05). However, the IFN-γ/IL-5 and IFN-γ/IL-10 ratios after re-stimulation with *L*. *major* F/T in group vaccinated with *L*. *lactis*-PpSP15-EGFP^cwa^ (G1) were higher than in both control groups in ST experiment (Fig 3E and 3F). However, in this experiment, the IL-17/IL-5 and IL-17/IL-10 ratios in G1 showed a significant difference with G3. Therefore, these results depicted a shift from Th2 to Th1 response and prevention of leishmaniasis improvement.

## Discussion

Long duration of the induced immune responses is one of the main concerns after a vaccine adminstration [43]. Here, we intended to establish protective immune responses after administration of *L*. *lactis* as a live non-pathogenic vaccine. It is obvious that *Leishmania* is a resilient parasite and through its unique gene regulation system can escape the host immune responses which has led to failure of its vaccine strategies so far [44]. Up to now, live vaccines (particularly leishmanization in high endemic area) have been shown to be more effective and stable in long-term, compared to other approaches such as the inactivated or the subunit vaccines [45]. Leishmanization and recently, live non-pathogenic microorganisms like *L*. *tarentolae*, are two successful examples of live delivery systems for expression of the protective proteins.

Other advantages of live vaccines are their lack of requirement to have adjuvants and be purified on the large scale and their low costs [46]. Non-pathogenic bacteria such as *L*. *lactis* are among the attractive live vehicles to express protein *in situ*. There are two major challenges when designing a live vaccine: choice of vehicle and selection of the immunogenic target. Localization of the expressed protein in the live cell is another problem. Among all non-pathogenic bacteria, *L*. *lactis* is not only safe in humans, but it also stimulates systemic immunity with heterologous antigen expression.

Previous studies have indicated that subcellular location of antigen expression exhibited that the cell wall attached antigens are more effective than cytoplasmic or secretory antigens to stimulate the immune system. This is because the proteins fold naturally and attach on the cell wall, and also they are not washed away during preparation of the live bacteria before immunization [47, 48].

In the present study, our aim was using *L*. *lactis* for stable expression of a prophylaxis protein (PpSP15) on the cell wall. For designing live delivery system, we selected an episomal system with several copies into the cell bacteria to avoid probability of any unintentional gene integration into the genome or integration under promoter with undesired activity [49]. Due to lack of specific antibody against PpSP15, the *egfp* gene was fused in downstream of *Ppsp*15 gene, to detect and confirm the protein expression. The Western blot analysis using anti-GFP antibody could confirm the expression of the protein as well. To check the location of PpSP15-EGFP protein expression, the cell wall fraction was isolated and this fraction was used as an antigen to be confirmed by ELISA and Western blot. Using Western blotting, the two specific bands related to EGFP (~27 kDa) and PpSP15-EGFP (~42 kDa) were observed in the membrane and the fractionated cell wall which could not be detected before the induction. Moreover, specific bands were obtained in the nonfragmented samples, which could be due to expression of the protein in the cytoplasm before its translocation to the cell wall [47].

Also, the results obtained from ELISA confirmed that EGFP is potentially expressed on the cell wall fraction. However, there was no powerful high absorbance for PpSP15 fused with EGFP. The reason is probably due to misfolding of EGFP after fusion with PpSP15; hence, the antibody could not identify PpSP15-EGFP. Therefore, to observe the expressed PpSP15-EGFP protein, we used TEM microscopy and interstingly, the secretion of a thick layer of PpSP15-EGFP expressed on the cell wall of *L*. *lactis-*PpSP15-EGFP^cwa^ could be recorded.

*L*. *lactis* bacteria are generally safe; however, due to certain safety concerns and also manipulation of the bacteria during the procedures, its toxicity should be checked in BALB/c mice. After i.v. single-dose injection of the recombinant bacteria, the mice were monitored daily. We observed no sign of weight loss, inflammation at injection sites, body temperature changes and abnormal behavior in these mice within 5 days compared to the mice which had received PBS as controls (S2 Fig).

Furthermore, after challenging the mice with *L*. *major*+SGH, the mice vaccinated with *L*. *lactis*-PpSP15-EGFP^cwa^ (G1) exhibited a smaller swelling size in their infected footpads. Footpad swelling differences between G1 and G3 were significant; however most of the time, this difference between G1 and G2 was not significant. Although there was no significant difference between G1 (*L*. *lactis*-PpSP15-EGFP^cwa^) and G2 (*L*. *lactis-*EGFP^cwa^) in both ST and LT experiments, the mean±SD in G1 was lower than in G2. However, immunogenicity of EGFP is a controversial subject [50–52]. However, foodpad swelling in mice by itself is not a valid indicator of the potential efficacy of vaccination or the infectivity rate [53]. Also, this regimen of immunization could control parasite propagation in the lymph nodes (LN). Importantly, the lowest parasite burden after the challenge in the LN at both short- and long-term experiments were observed in G1 versus control groups (G2 and G3). As shown by Zahedifard et al. at 2014, PpSP15 as a powerful antigen in different modalities such as DNA vaccine or expressed in *L*. *tarentolae*, has led to the least parasite burden in the LN after infection with *L*. *major* [9].

Additionally, our results indicated for the first time that homologous prime-boost vaccination with *L*. *lactis*-PpSP15-EGFP^cwa^ enhanced the early production of IFN-γ, IL-17 and reduced IL-10 and IL-5, which could alleviate CL due to *L*. *major*. The cellular immune responses through Th1 is responsible for generating a protective immunity against *Leishmania* and based on our data, this response is stable for 6 months. The significant differences between G1 and other groups to produce IFN-γ and IL-17 points out to the role of specific antigen (i.e. PpSP15 protein). Moreover, in spite of the unkown role of IL-17, these results show that the increasing IFN-γ is accompanied with increasing IL-17. Although the IL-17 effect on the immune response is still controversial [54–57], our results are consistent with several other studies that have shown that IL-17 might help vaccine-induced protection synergistically, along with IFN-γ [57–61]. In our results, although the levels of IFN-γ and IL-17 in ST were higher than in LT experiment, a lower amount of these cytokines could show a significant difference with G3 control. As reported, heterologous prime-boost vaccination with DNA PpSP15 or homologous prime-boost with *L*. *tarentolae* expressing PpSP15 alone or combined with CpG ODN as an adjuvant led to Th1 response in BALB/c mice after infection with *L*. *major* [9, 10]. A live vaccination using *L*. *tarentoale*-PpSP15-EGFP generated the most IL-17 beside IFN-γ in ST (8 weeks) after immunization [10]. As previously noted, there are IL-10 negative impacts for generating the Th1 immune response [62], and it has been reported that IL-10 inhibits the production of IL-17 [63], indicating a reversal effect between IL-10 and IL-17, which was also evident in our data. In particular, the relation between salivary proteins and IL-17 response is still debatable because an immunity role for IL-17 to cause anti-saliva proteins immunity is suggested [64].

IL-10 has an important regulatory role on Th1 through inhibition of macrophages to perform phagocytosis and killing of the parasite [65]. In the short-term experiment, after infection with the parasite and stimulation with F/T of the parasite, the IL-10 level in G1 (immunized with *L*. *lactis*-PpSP15-EGFP^cwa^) was decreased by 2.3x (~43%). Also, the secretion level of IFN-γ and IL-17 in G1 were increased by 2.71x and 1.18x, respectively. Generally, in contrast with G3 (non-immunized), immunized G1 was associated to significantly control the disease through inhibition of ~55% parasite propagation in LNs. However in both experiments, after stimulation with *L*. *major* F/T, ratios of IFN-γ and IL-17 to IL-5 and IL-10 in G1 are more than 1 (ranging between 5–16), indicating a dominant Th1 response.

Of course, we cannot ignore the impact of *L*. *lactis* on the immune system because it was used in its live form and it may also induces innate and adaptive immunities [66, 67]. This lack of a significant difference may be due to the adjuvant properties of *L*. *lactis* [66, 67]. Hence, we sometimes observed that the reaction of G2 were not different from G1 that was used as a main immunized group.

Besides, the results of LT memory suggested the generation of the immune responses by *L*. *lactis*-PpSP15-EGFP^cwa^. We observed that six months after the immunization, the mice responses against *L*. *major* plus SGH was very similar to the ST experiment, and patterns of the produced cytokines were comparable. In addition, cytokine pradigm demonstrated a continuous Th1 response against *L*. *major*. Therefore, expression of PpSP15 on the cell wall of *L*. *lactis* could establish the immune response toward LT protection, most likely, because *L*. *lactis*-PpSP15-EGFP^cwa^ remained or proliferated in mice body and was continuously exposed to the immune responses. On the other hand, it may be due to the creation of memory T cells, which in secondary encounter could cause a protective response. However, higher parasite burden and lower proinflammatory cytokines could be related to the unstability of the bacetria in LT.

In other studies, mice immunized with the cell wall and secretory form of A2-expressing *L*. *lactis* after challenge with *L*. *donovani*, not only showed the lowest parasite burden in comparison with other groups, particularly cytoplasm form of A2 expression, but they also exhibited increased IFN-γ levels and also decreased IL-10 levels that are the hallmark of a Th1 immune response [47]. Furthermore, immunization of mice with the cell wall and secretory form of LACK expressing *L*. *lactis* has shown the high level of IFN-γ /IL-10 ratio which as said above is an indication of a Th1 immune response, necessary to combat *Leishmania major* [68].

### Conclusion

In our study, similar to other reports, the decreasing parasite burden and significant increase in IFN-γ /IL-5, IFN-γ /IL-10 and also in IL-17/IL-5 and IL-17/IL-10 ratios in immunized BALB/c mice with *L*. *lactis*-PpSP15-EGFP^cwa^ after the challenge indicated that membrane-associated PpSP15 on the *L*. *lactis* as an applicable live vector for human studies could develop a strong Th1-immune response. The combination of findings provides some support for *L*. *lactis*-PpSP15-EGFP^cwa^ to be utilized as an experimental live vaccine against CL. In future investigations, it can be considered to use a combination of PpSP15 with one of the parasite antigens expressed in *L*. *lactis* as a live vaccine for specific protective immune responses against *Leishmania* species.

## Supporting information

S1 Fig Gene cloning and confirmation of expressed proteins in *L. lactis*. (A) Schematic figures of two plasmid constructs, pNZ8121-*Ppsp15-egfp* and pNZ8121-*egfp*. (B) Enzymatic confirmation of genes using *Xba*I/*Eco*RV restriction enzymes (Lane 1: *egfp* gene, ~750 bp; Lane 2: *Ppsp15-egfp*, ~1130 bp; Lane 3: the molecular weight marker (1kb). (C) Western blot analysis using anti-GFP antibody showed two specific bands (~27 and ~42 kDa related to EGFP and PpSP15-EGFP, respectively). rEGFP (~27 KDa) was used as a positive control. No; non-induced recombinant bacteria, BI; before induction, ON; overnight. (D) Microscopy images with light (right) and fluorescent (left) microscope. At 3 h or overnight (has been shown just for *L*. *lactis*-PpSP15-EGFP^cwa^) after induction, both recombinant bacteria were monitored to confirm EGFP protein expression. (E) Flow cytometry analysis using FACS caliber also revealed that the intensity of EGFP in the *L*. *lactis* wild-type (left) as a negative control was 0.88% and in the recombinant *L*. *lactis*-EGFP^cwa^ (middle) as a positive control was 72.11%, and in the *L*. *lactis*-PpSP15-EGFP^cwa^ (right) was 31.86%. This evaluation was done many times and results of one of the assays have been shown here.
(TIF)

S2 Fig Toxicity assay of recombinant *L*. *lactis* in BALB/c mice. Different groups of BALB/c mice (3 mice/group) were injected as i.v. into the tail vein with different bacteria (*L*. *lactis*-PpSP15-EGFP^cwa^ or *L*. *lactis*-EGFP^cwa^) or PBS as a control. Mice weight until 120 h after injection was measured using a digital scale (OHAUS).
(TIF)

S1 Table Vaccination regimens in different mice groups. (TIF)
