# Supplementary figures and images for: Lactococcus lactis expressing sand fly PpSP15 salivary protein confers long-term protection against Leishmania major in BALB/c mice
Source: PLoS Negl Trop Dis. 2020 Jan 3;14(1):e0007939. doi: 10.1371/journal.pntd.0007939 (PMC6941807; doi:10.1371/journal.pntd.0007939)

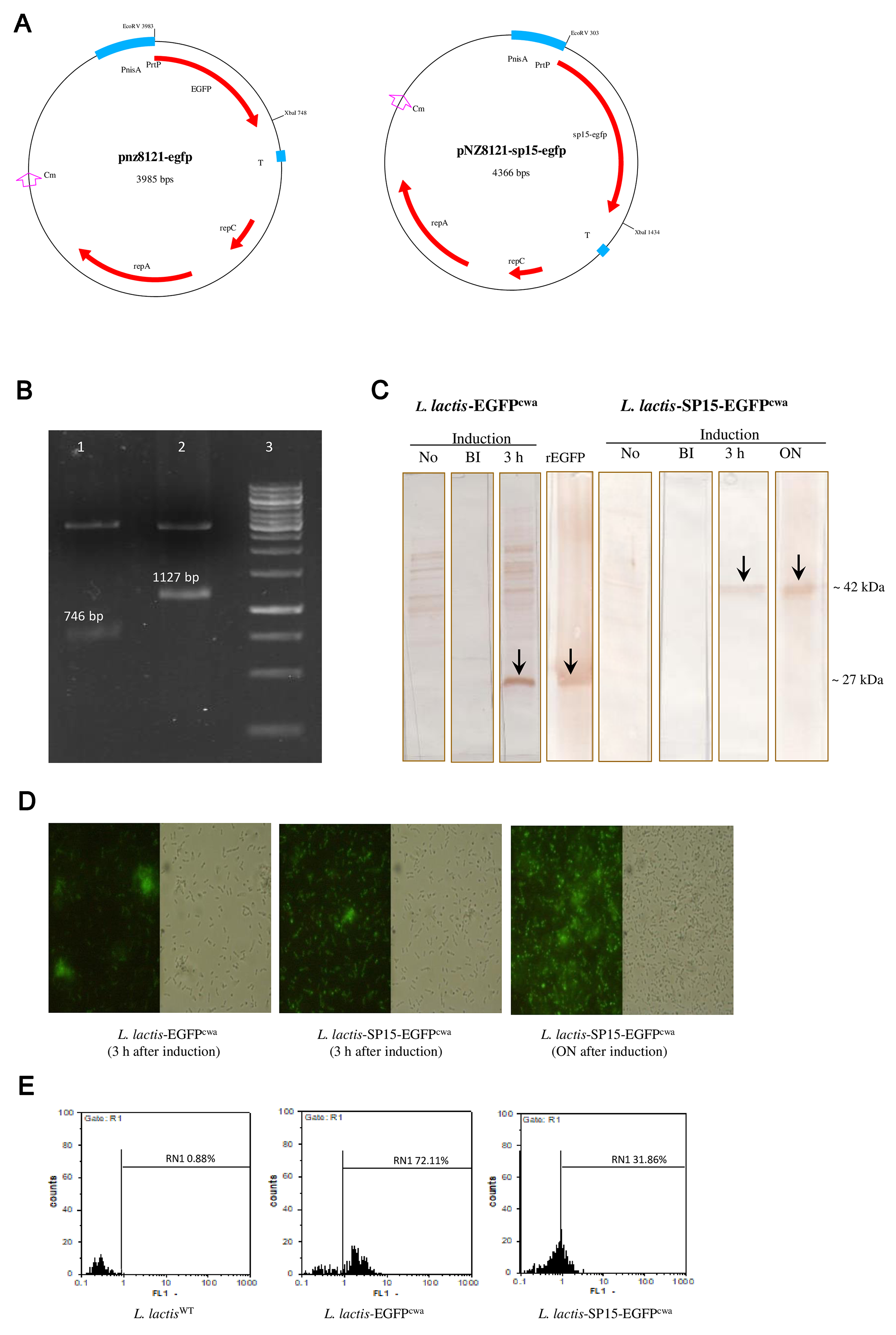

Supplement: S1 Fig — (A) Schematic figures of two plasmid constructs, pNZ8121-Ppsp15-egfp and pNZ8121-egfp. (B) Enzymatic confirmation of genes using XbaI/EcoRV restriction enzymes (Lane 1: egfp gene, ~750 bp; Lane 2: Ppsp15-egfp, ~1130 bp; Lane 3: the molecular weight marker (1kb). (C) Western blot analysis using anti-GFP antibody showed two specific bands (~27 and ~42 kDa related to EGFP and PpSP15-EGFP, respectively). rEGFP (~27 KDa) was used as a positive control. No; non-induced recombinant bacteria, BI; before induction, ON; overnight. (D) Microscopy images with light (right) and fluorescent (left) microscope. At 3 h or overnight (has been shown just for L. lactis-PpSP15-EGFPcwa) after induction, both recombinant bacteria were monitored to confirm EGFP protein expression. (E) Flow cytometry analysis using FACS caliber also revealed that the intensity of EGFP in the L. lactis wild-type (left) as a negative control was 0.88% and in the recombinant L. lactis-EGFPcwa (middle) as a positive control was 72.11%, and in the L. lactis-PpSP15-EGFPcwa (right) was 31.86%. This evaluation was done many times and results of one of the assays have been shown here. (TIF) [file pntd.0007939.s001.tif]

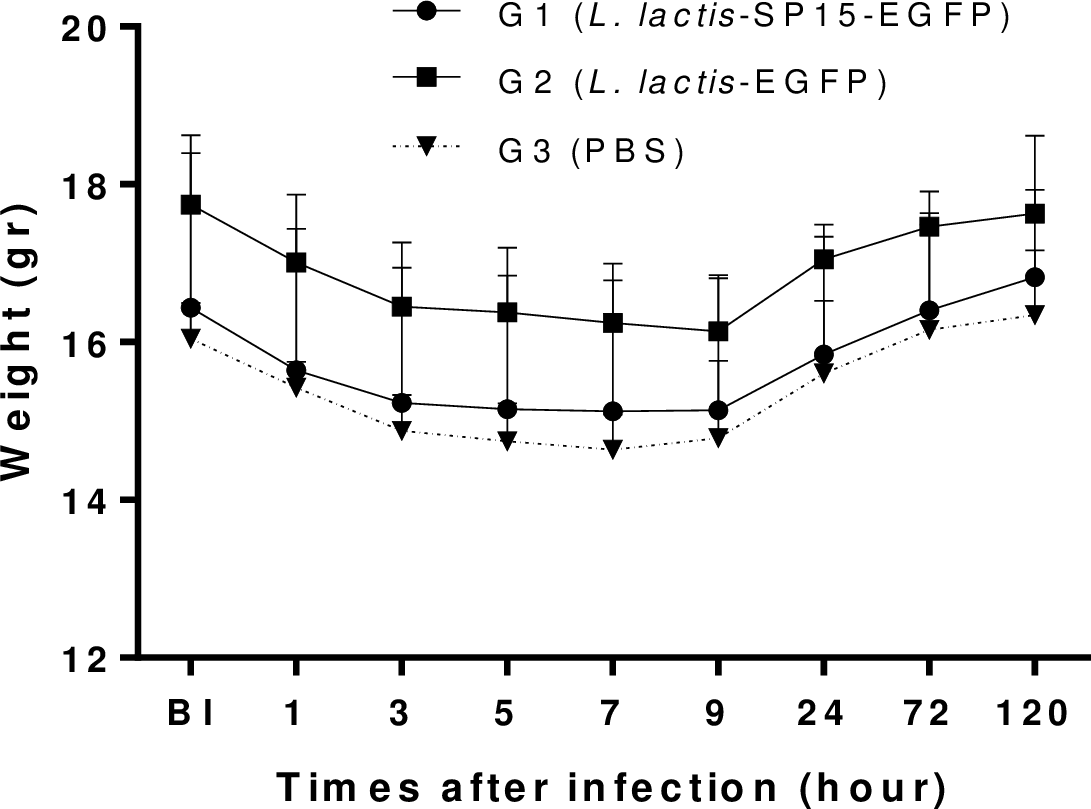

Supplement: S2 Fig — Different groups of BALB/c mice (3 mice/group) were injected as i.v. into the tail vein with different bacteria (L. lactis-PpSP15-EGFPcwa or L. lactis-EGFPcwa) or PBS as a control. Mice weight until 120 h after injection was measured using a digital scale (OHAUS). (TIF) [file pntd.0007939.s002.tif]

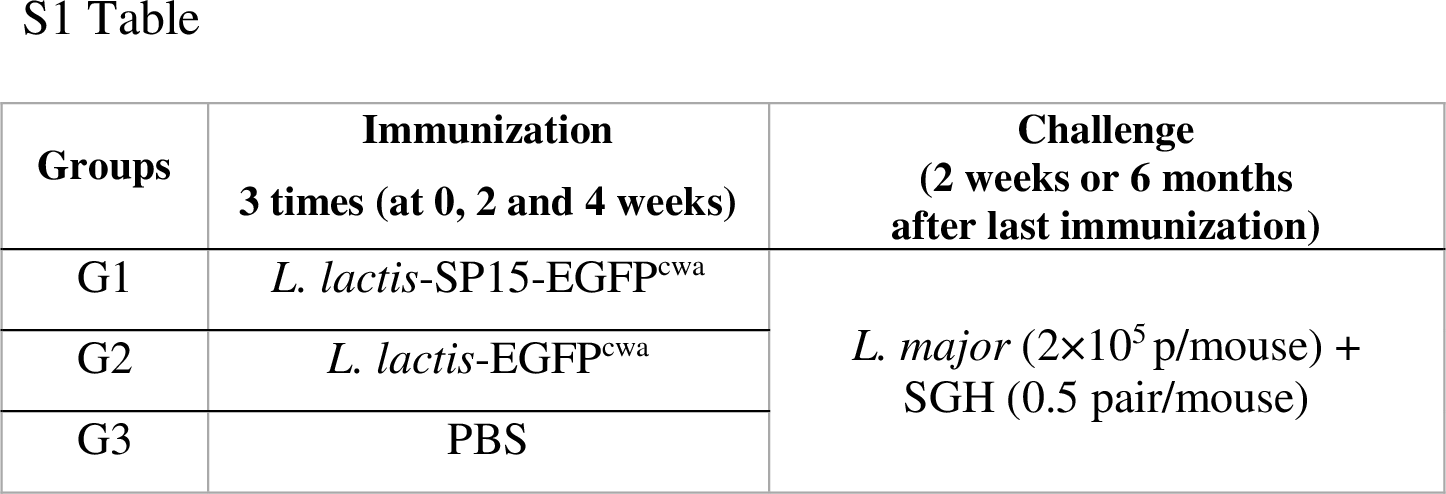

Supplement: S1 Table — (TIF) [file pntd.0007939.s003.tif]
